# The Worth of Words

**Published:** 1904-04

**Authors:** 


THE WORTH OF WORDS. By Dr. Ralcy H. Bell. Hinds and Noble, Publishers, 31-33-35 W. 15th Street, New York.

Dr. Bell has produced a very readable book, especially for those who desire to cultivate purity of speech. They will find the correct form of many errors ot speech that creep into the conversation and writing of many who should know better. The book is pretty in a peculiar style that engages the attention and serves to enliven an otherwise dull subject.

A great mass of words and phrases have been collected and analyzed.

The book is well worth perusal and purchase.

